# 5-HMF attenuates inflammation and demyelination in experimental autoimmune encephalomyelitis mice by inhibiting the MIF-CD74 interaction: Role of 5-HMF in EAE mice

**DOI:** 10.3724/abbs.2023105

**Published:** 2023-07-10

**Authors:** Dongsheng Guan, Yingxia Li, Yinglin Cui, Huanghong Zhao, Ning Dong, Kun Wang, Deqi Ren, Tiantian Song, Xiaojing Wang, Shijie Jin, Yinghe Gao, Mengmeng Wang

**Affiliations:** 1 Department of Neurology the Second Clinical Medical College Henan University of Traditional Chinese Medicine Zhengzhou 450002 China; 2 The College of Basic Medicine Henan University of Traditional Chinese Medicine Zhengzhou 450046 China; 3 Department of Pharmacy the Second Clinical Medical College Henan University of Traditional Chinese Medicine Zhengzhou 450002 China

**Keywords:** multiple sclerosis, experimental autoimmune encephalomyelitis, 5-hydroxymethyl-2-furfural, M2 polarization, inflammation

## Abstract

The neuroprotective role of 5-hydroxymethyl-2-furfural (5-HMF) has been demonstrated in a variety of neurological diseases. The aim of this study is to investigate the effect of 5-HMF on multiple sclerosis (MS). IFN-γ-stimulated murine microglia (BV2 cells) are considered a cell model of MS. With 5-HMF treatment, microglial M1/2 polarization and cytokine levels are detected. The interaction of 5-HMF with migration inhibitory factor (MIF) is predicted using online databases. The experimental autoimmune encephalomyelitis (EAE) mouse model is established, followed by a 5-HMF injection. The results show that 5-HMF facilitates IFN-γ-stimulated microglial M2 polarization and attenuates the inflammatory response. According to the network pharmacology and molecular docking results, 5-HMF has a binding site for MIF. Further results show that blocking MIF activity or silencing *CD74* enhances microglial M2 polarization, reduces inflammatory activity, and prevents ERK1/2 phosphorylation. 5-HMF inhibits the MIF-CD74 interaction by binding to MIF, thereby inhibiting microglial M1 polarization and enhancing the anti-inflammatory response. 5-HMF ameliorates EAE, inflammation, and demyelination *in vivo*. In conclusion, our research indicates that 5-HMF promotes microglial M2 polarization by inhibiting the MIF-CD74 interaction, thereby attenuating inflammation and demyelination in EAE mice.

## Introduction

Multiple sclerosis (MS) is a chronic inflammatory disease of the central nervous system (CNS) characterized by inflammatory cell infiltration, demyelination, and subsequent formation of sclerotic plaques in brain tissue [ 1, 2] . It is a common disabling neurodegenerative disease of young adults, and lesions are concentrated in the brain and spinal cord [3]. The prevalence of MS is high worldwide, but the pathogenic mechanisms are not fully understood [4].

Experimental autoimmune encephalomyelitis (EAE) is the most widely used preclinical murine model of human MS [5], and studies have shown that microglial activation plays a critical role in EAE [ 6, 7] . Microglia are tissue-resident macrophages in the CNS [8]. In the early stage of EAE in mice, microglia in the CNS are transformed to the M1 phenotype, leading to tissue damage and progression of EAE. In the later stage, the M2 phenotype dominates, which plays a role in alleviating inflammation and promoting tissue repair. Therefore, inhibition of microglial M1 polarization and induction of M2 polarization can be considered therapeutic strategies to alleviate EAE [9].

Bu-Shen-Yi-Sui capsule, a traditional Chinese herbal medicine, has a significant effect on improving the symptoms of EAE mice and MS patients, and its mechanism involves the promotion of M2 polarization [ 10, 11] . 5-Hydroxymethylfurfural (5-HMF), the main ingredient of Bu-Shen-Yi-Sui capsules, is a kind of aldehyde compound with a furan ring structure and can cross the blood-brain barrier [ 12, 13] . Recent studies have suggested that 5-HMF is a novel agent in traditional Chinese medicine with antioxidant and brain-protective effects. For example, Zhang *et al*. [12] suggested that 5-HMF can protect hippocampal neurons from damage caused by a high concentration of corticosterone. Ya *et al*. [14] claimed that 5-HMF alleviates striatal oxidative damage after transient global cerebral ischemia. In addition, 5-HMF has been reported to inhibit the activation of LPS-induced macrophages and reduce the secretion of inflammatory factors [15]. However, it remains unclear whether 5-HMF improves MS symptoms by regulating microglial polarization.

Migration inhibitory factor (MIF) is a small secretory protein composed of three homotrimers with the same primary sequence that was first discovered as a lymphokine that prevents macrophages from migrating out of the capillary [ 16, 17] . Subsequent studies confirmed that MIF can be secreted by a variety of cells, including monocytes/macrophages [18], eosinophils [19], and neutrophils [20]. During neuroinflammation, macrophages/microglia are the major sources of MIF in the CNS [21]. MIF is currently considered a proinflammatory cytokine involved in various immune and inflammatory responses. It has been shown that MIF plays a key role in inflammation, the immune response, and pathogenesis, especially in tumor pathogenesis and the neuroendocrine axis, by forming complexes with receptors such as CD74, CXCR4, CXCR2, and CD44 [ 22– 24] . The interaction of MIF with CD74 is critical for accelerating the progression of EAE [ 25, 26] . An early study found that targeting the MIF-CD74 interaction affects the inflammatory response of obesity-related adipose tissue by mediating M1 macrophage polarization [27]. In addition, some studies have confirmed that MIF activates extracellular signal-regulated kinase 1/2 (ERK1/2) by binding to CD74, which promotes the secretion of proinflammatory mediators and affects the M1 polarization of microglia [ 28– 30] .

Based on the above evidence, we speculated that 5-HMF might regulate microglial polarization by affecting the MIF-CD74 interaction, thereby attenuating EAE in mice and MS in patients. This is the first report to demonstrate the function of 5-HMF in inflammation and demyelination in MS.

## Materials and Methods

### Cell culture, transfection, and treatment

BV2 murine microglia were obtained from the Institute of Basic Medicine, Chinese Academy of Medical Sciences (Beijing, China). Cells were cultured in DMEM (Thermo Fisher Scientific, Waltham, USA) containing 10% fetal bovine serum (FBS; Gibco, Waltham, USA) at 37°C in the presence of 5% CO _2_. The medium was changed every 2 days.

When the cells reached approximately 70%–80% confluence, they were prepared for transfection. Small interfering RNAs (siRNAs), including si-CD74 and si-NC, were purchased from GenePharma (Shanghai, China). They were transfected into BV2 cells using Lipofectamine 2000 (Invitrogen, Waltham, USA) for 24 h according to the manufacturer’s instructions. After transfection, cells were treated with IFN-γ (100 ng/mL) for 24 h. The siRNA sequences were as follows: si-CD74 sense, 5′-ACUAAUGGGUCAGAAAUGGGG-3′, and antisense, 5′-CCAUUUCUGACCCAUUAGUAG-3′; si-NC sense, 5′-UUCUCCGAACGUGUCACGUTT-3′, and antisense, 5′-ACGUGACACGUUCGGAGAATT-3′.

For M1 polarization induction, BV2 cells were stimulated with IFN-γ (100 ng/mL) for 24 h [31]. For 5-HMF treatment, BV2 cells were stimulated with IFN-γ (100 ng/mL) for 2 h, followed by 5-HMF (10, 30, and 100 μg/mL) treatment for 24 h [32]. For MIF inhibitor (ISO-1) treatment, BV2 cells were stimulated with IFN-γ (100 ng/mL) for 2 h, followed by ISO-1 (50 μM) treatment for 24 h [33]. For the recombinant MIF (rMIF) and ERK1/2 inhibitor (PD98059) treatments, rMIF (100 ng/mL) was added to BV2 cells and maintained for 24 h [29], then PD98059 (10 μM) was added and incubated for 24 h [34].

### Isolation of mouse primary microglia

The coverslips were cut into 0.5× 0.5 cm pieces, washed, and dried. They were then soaked in 5% hydrochloric acid for 30 min, rinsed in tap water for 20 min, and rinsed in triple-distilled water overnight before being transferred to 95% alcohol for storage. The coverslips were placed in 24-well cell culture plates. Culture dishes and small slides were coated with 0.01% L-polylysine solution and incubated at 37°C for 4 h or overnight at room temperature. The culture dishes and small slides were rinsed with sterilized deionized water. Brain tissues of SPF mice were collected within 24 h of the neonatal period. The cerebral cortex was isolated and cut into approximately 1 mm ^3^ tissue pieces. The tissues were digested with 0.125% trypsin. After discarding the supernatant, the complete inoculum was added to complete the digestion and rinsed twice. The tissues were gently aspirated with a pipette. The suspension was collected into a new centrifuge tube and centrifuged at 1000 *g* for 10 min at 4°C, and the supernatant was discarded. The complete culture broth was added to centrifuge tubes and filtered through 200-mesh stainless steel mesh. Samples were inoculated into 25-cm cell culture flasks and incubated at 37°C in a 5% CO _2_ incubator. The medium was changed after 24 h and every 3 d thereafter, and cell growth and survival were observed under a microscope.

### Western blot analysis

BV2 cells and spinal cord segments (1 cm) were washed with PBS and lysed with RIPA buffer at 4°C for 30 min. Lysates were centrifuged at 12,000 *g* for 20 min at 4°C to remove the insoluble pellets. Protein concentration was determined using a BCA kit (Beyotime, Shanghai, China). Equal amounts of protein were separated by 10% SDS-PAGE and then transferred to polyvinylidene difluoride (PVDF) membranes (Millipore, Temecula, USA). After incubation with 5% skim milk for 1 h, the membranes were incubated with primary antibodies including anti-iNOS (1:1000, ab283655; Abcam, Cambridge, UK), anti-Arg-1 (1:1000, ab233548; Abcam), anti-MIF (1:1000, PA5-27343; Invitrogen), anti-ERK1/2 (1:10000, ab184699; Abcam), anti-p-ERK1/2 (1:2000, 4370T; Cell Signaling Technology, Danvers, USA), anti-CD74 (1:1000, ab289885; Abcam), and anti-GAPDH (1:2000, ab8245; Abcam). The next day, the membranes were incubated with the corresponding HRP-conjugated goat anti-rabbit IgG (1:3000, ab205718; Abcam) or goat anti-mouse IgG (1:3000, ab6789; Abcam) secondary antibodies for 2 h at room temperature. The bands were then detected using ECL reagents (Beyotime, Beijing, China).

### Flow cytometry

After treatment with IFN-γ and 5-HMF, BV2 cells were harvested for flow cytometry detection as previously described [35]. Briefly, cells were fixed, permeabilized, and incubated with rabbit anti-mouse iNOS (ab283668; Abcam) or sheep anti-mouse Arg-1 (10 μL, IC5868P; R&D Systems, Minneapolis, USA) at 4°C for 30 min. After being washed with the specified washing buffer, cells were harvested and detected with a FACS Caliber instrument (BD Biosciences, San Jose, USA), and the relative mean fluorescence in each group was analysed using Flow Jo software (version 10.0; Tree Star, Ashland, USA).

### Enzyme-linked immunosorbent assay (ELISA)

The ELISA kits for TNF-α (MTA00B; R&D Systems), IL-10 (DY417; R&D Systems) and MIF (DY1978; R&D Systems) were used to measure the levels of TNF-α and IL-10 in BV2 cell supernatants and mouse serum, and the level of MIF in BV2 cell supernatants according to the manufacturer’s protocols and previous descriptions [36]. Before detection, the kits were kept at room temperature for 30 min, and the samples were diluted 5-fold. Then, the measurement was performed, and the absorbance was detected at 450 nm using a microplate reader (BioTek, Winooski, USA). The concentrations of TNF-α, IL-10, and MIF were calculated from the absorbance values and the standard curve.

### Coimmunoprecipitation (Co-IP) assay

To confirm the interaction between CD74 and MIF, 10 μL of anti-HA beads (Beyotime, Beijing, China) were added and incubated with BV2 cell lysate or spinal cord tissue containing HA or flag-tagged CD74 and MIF at 4°C overnight. BV2 cell lysate or spinal cord tissue was incubated with anti-CD74 or anti-MIF and 10 μL protein A/G agarose (Pierce, Rockford, USA) at 4°C overnight. The precipitates were washed and resuspended in a 5× loading buffer. Finally, the samples were boiled in a water bath for 10 min and subject to western blot analysis with antibodies against CD74 or MIF.

### Quantitative real-time PCR

Total RNA was extracted from BV2 cells using TRIzol reagent (Invitrogen). The mRNA was reverse transcribed using the ReverTra qPCR RT Kit (Toyobo, Osaka, Japan). The CD74 mRNA level was measured with the SYBR Premix Ex TaqII kit (Takara, Dalian, China) using a 7300 real-time PCR system (Thermo Fisher Scientific). The CD74 mRNA level was normalized to the expression of *β-actin* and calculated using the 2 ^–ΔΔCt^ method.

### Animal model

Eighteen female 10–12-week-old C57BL/6 mice were purchased from Shanghai SLAC Laboratory Animal Co., Ltd (Shanghai, China). They were housed under SPF conditions with food and water ad libitum and a 12-h light/dark cycle. The animal experiments were approved by the Ethics Committee of the Second Clinical Medical College, Henan University of Traditional Chinese Medicine. To establish the EAE model, mouse myelin oligodendrocyte glycoprotein peptide 35–55 (MoG _35–55_, 300 μg/mouse, YRNGK; Maokang Biotechnology, Shanghai, China) was completely dissolved in complete Freund’s adjuvant (Sigma-Aldrich, St Louis, USA) and mixed with attenuated Mycobacterium tuberculosis H37R (400 μg/mouse; BD Difco, Solon, USA). The above mixture (100 μL) was injected subcutaneously into the upper dorsal flank of the mice. Meanwhile, mice received 300 ng of pertussis toxin (List Labs, Campbell, USA) by intraperitoneal injection, and this injection was repeated 48 h later [ 37, 38] . For the 5-HMF treatment, mice received 5-HMF (12 mg/kg) by intraperitoneal injection from day 3 to day 27 after the subcutaneous immunization [ 14, 37] . In this process, the neurological function scores and body weight were blindly assessed daily by two investigators. The score was evaluated as follows: 0, healthy; 1, waddling gait without a tail reflex; 2, paresis of the hind limbs with a limp tail (ataxia); 3, complete paralysis of one limb; 4, tetraparalysis; and 5, death.

### Histological analysis

On day 28 after the subcutaneous immunization, the spinal cords were removed from mice after anaesthetization with 10% chloral hydrate and fixed in 4% paraformaldehyde. Spinal cord tissues were sectioned into 10-μm sections using a cryostat microtome (CM3050S; Leica, Wetzlar, Germany). Pathological changes in the spinal cords were detected by Luxol fast blue (LFB) and hematoxylin/eosin (H&E) staining [ 39, 40] . For LFB staining, the sections were stained in LFB at 56°C for 16 h. Excess blue staining solution was removed with 95% ethanol, followed by differentiation performed in lithium carbonate for 15 s. After being washed with 80% alcohol, the sections were dehydrated and mounted with neutral balsam. For H&E staining, the sections were stained in hematoxylin solution for 5 min, followed by water for 1 min, and in eosin solution for 10 min. The sections were then differentiated in 1% hydrochloric acid ethanol for 10 s. The sections were imaged under a light microscope (DM4000B; Leica).

For the statistical analysis, three histological sections were selected for each mouse, and the average scores were analysed. The demyelination score was assessed as follows: 0, none; 1, rare foci; 2, a few areas of demyelination; and 3, large or confluent areas of demyelination. The inflammatory infiltration score was evaluated as follows: 0, no infiltrating cells; 1, few scattered infiltrating cells; 2, inflammatory infiltration around blood vessels; and 3, extensive perivascular cuffing with extensive infiltration [41].

### Immunofluorescence staining

Lumbar spinal cord tissue sections were washed with PBS and blocked in 5% BSA for 30 min. The sections were then incubated with primary antibodies, including anti-Iba1 (1 μg/mL, ab178846; Abcam), anti-iNOS (1:50, ab283655; Abcam), and anti-CD206 (1:100, CL488-60143; Proteintech, Rosemont, USA). After wash with PBS, sections were incubated with the appropriate secondary antibodies for 3 h at room temperature. DAPI solution (Sigma-Aldrich) was used for nuclear staining for 5 min. Fluorescence images were visualized and captured under a Nikon 300 fluorescence microscope (Nikon, Tokyo, Japan). Immunofluorescence staining with anti-iNOS (1:50, ab283655; Abcam) and anti-Arg-1 (1:400, #43279; Cell Signaling Technology) was performed in BV2 cells.

### Statistical analysis

Data were expressed as the mean±standard error of the mean (SEM) and analysed using SPSS 13.0 software. Statistical significance was determined by one-way analysis of variance (ANOVA) followed by Tukey’s *post hoc* test. *P*<0.05 was considered to be statistically significant.

## Results

### 5-HMF facilitates IFN-γ-stimulated microglial M2 polarization and attenuates the inflammatory response

**Figure 1:**
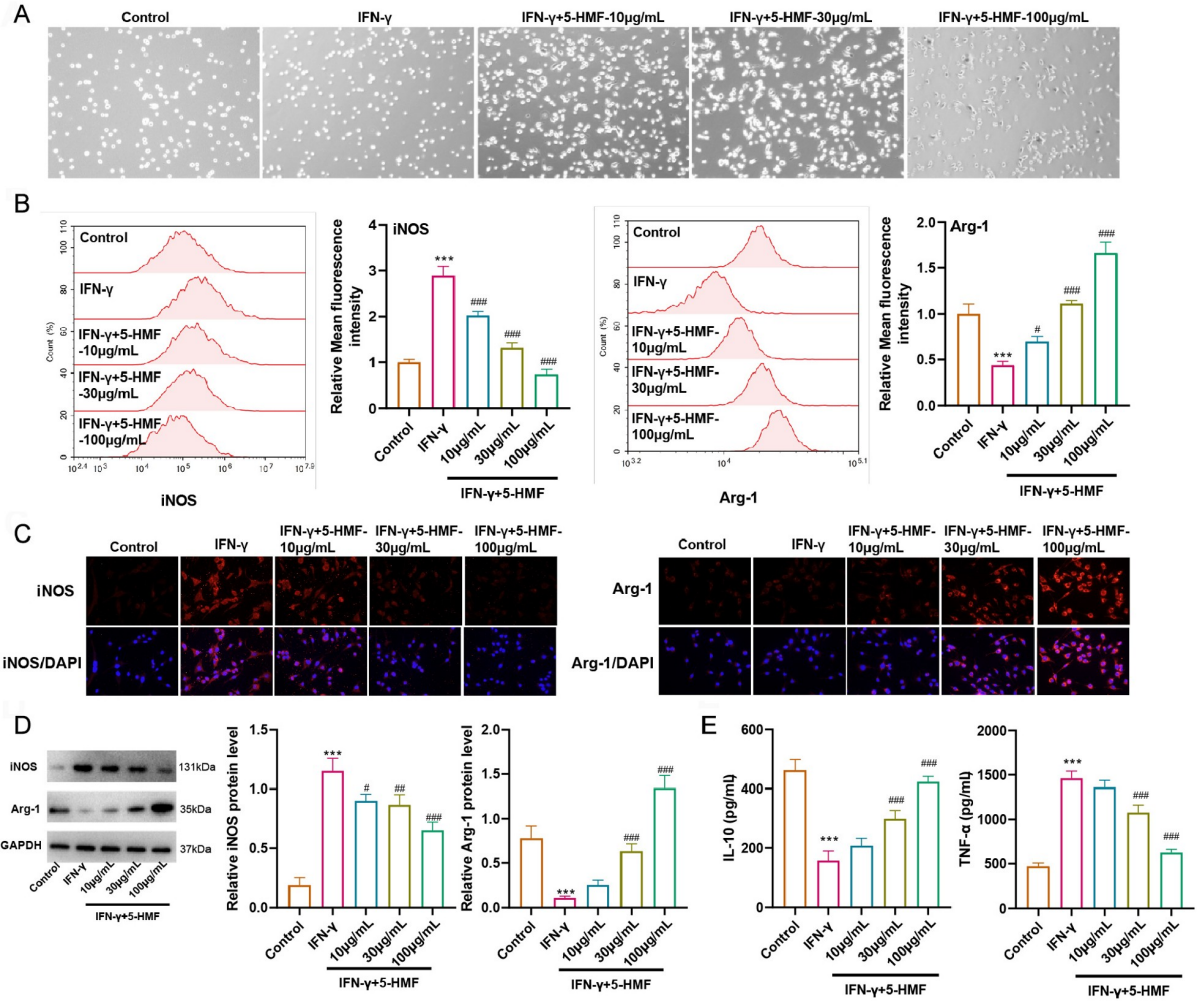
Figure 1 5-HMF facilitates IFN-γ-stimulated microglial M2 polarization and attenuates the inflammatory response BV2 cells were stimulated with IFN-γ (100 ng/mL) for 2 h followed by 5-HMF treatment at concentrations of 10, 30, and 100 μg/mL. Cells were grouped as follows: control, IFN-γ, and IFN-γ+5-HMF (10, 30, and 100 μg/mL). (A) Morphologic observations of BV2 cells. (B) Flow cytometric analysis. iNOS-positive cells and Arg-1-positive cells were evaluated. The mean fluorescence intensity of each condition was analysed. (C) Immunostaining against iNOS and Arg-1 antibodies. (D,E) The protein levels of iNOS and Arg-1 in cells and the concentrations of TNF-α and IL-10 in cell supernatants were measured by western blott analysis and ELISA. GAPDH was used as an internal control. Statistical analysis was performed by one-way ANOVA followed by Tukey’s post hoc test. Each experiment was independently performed in triplicate. *** P<0.001 vs Control; #P<0.05, ##P<0.01, ###P<0.001 vs IFN-γ.

A previous study demonstrated that IFN-γ-stimulated microglia could induce proinflammatory M1 macrophage polarization [42]. Some reports confirmed that 5-HMF inhibited LPS-induced macrophage activation, reduced the secretion of inflammatory factors, and exerted protective effects on hippocampal neurons [ 12, 15] . To clarify the role of 5-HMF in the central nervous system (CNS), an *in vitro* inflammatory model was induced with BV2 cells via IFN-γ stimulation, followed by 5-HMF treatment at the indicated time points. Morphological observations revealed that the normal or IFN-γ-stimulated cells were round and elongated with an irregular shape, while long dendritic formations were gradually stimulated along with increasing concentrations of 5-HMF, suggesting the induction of M2 polarization ( Figure 1A). The results of flow cytometry and immunostaining showed that IFN-γ significantly increased the number of iNOS-positive cells, while increasing doses of 5-HMF gradually decreased this change. In contrast, IFN-γ markedly decreased the number of Arg-1-positive cells, whereas increasing doses of 5-HMF gradually enhanced this level ( Figure 1B,C). In addition, according to the western blot analysis and ELISA results, 5-HMF treatment downregulated the iNOS protein level in IFN-γ-stimulated cells and reduced the TNF-α level in the supernatants. The Arg-1 level in IFN-γ-stimulated cells and the IL-10 level in supernatants showed the opposite trend ( Figure 1D,E). Collectively, these data suggested that 5-HMF facilitated IFN-γ-induced M2 polarization and enhanced the anti-inflammatory response.

### Binding of 5-HMF with MIF validated by network pharmacology-based prediction and molecular docking

**Figure 2:**
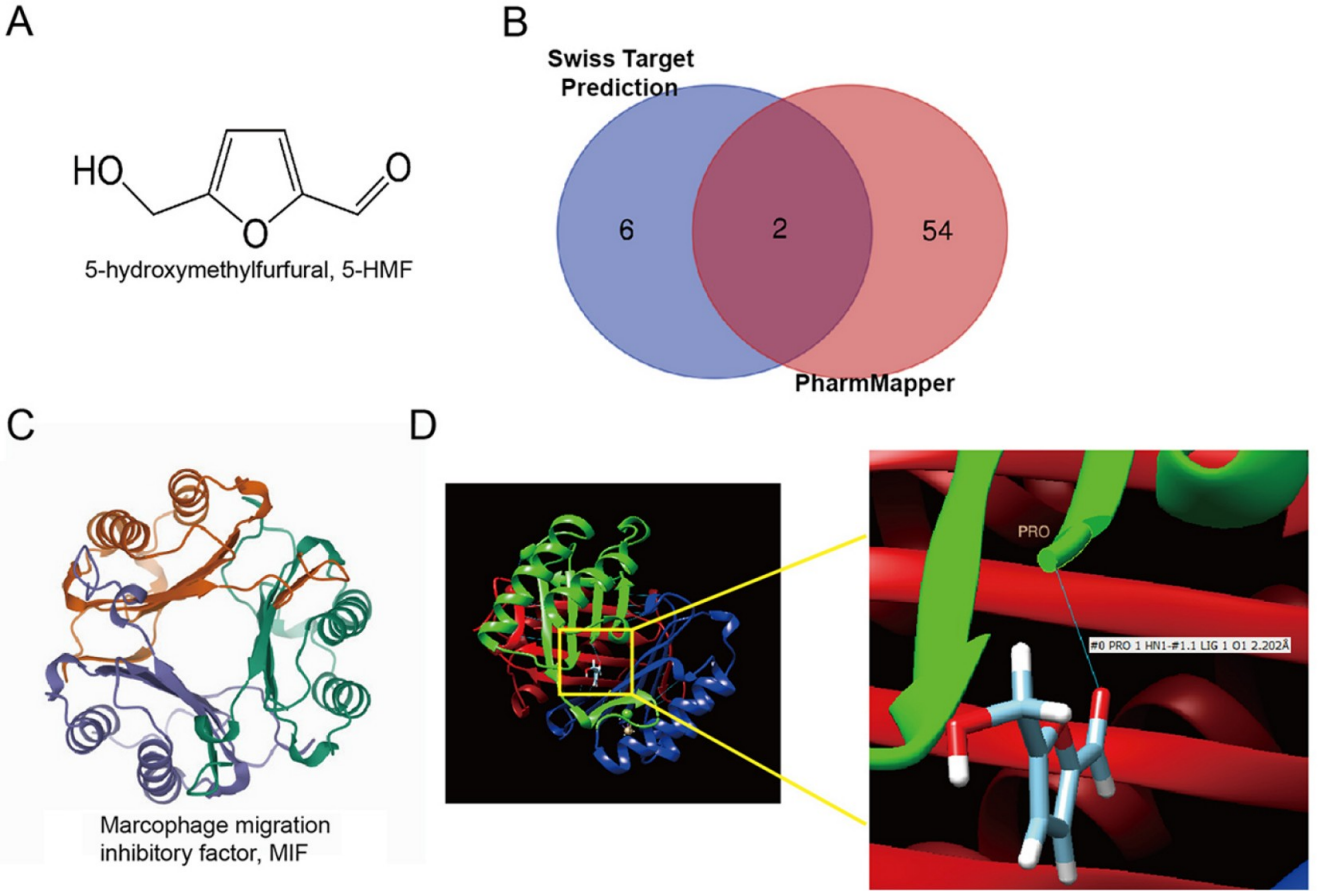
Figure 2 The binding of 5-HMF with MIF was validated by network pharmacology-based prediction and molecular docking (A) Chemical structure of 5-HMF, which was downloaded from the PubChem database ( https://pubchem.ncbi.nlm.nih.gov/). (B) Predicted targets of 5-HMF are displayed in a Venn diagram. The targets of 5-HMF were predicted by the Swiss Target Prediction database ( http://swisstarget prediction.ch/) and the PharmMapper database ( http://lilab-ecust.cn/pharmmapper/). (C) 3D structure of MIF. (D) The possible molecular docking results between 5-HMF and MIF were obtained from the SwissDock online server. Combined using UCSF Chimera software, the optimal position information of the binding site of 5-HMF and MIF was obtained. #0 is a meaningless label; PRO1 represents proline at position 1; HN1 represents hydrogen bonding; #1.1 is a meaningless label; LIG1 represents ligand; O1 represents the oxygen at position 1 of the compound; 2.202 Å represents the hydrogen bonding force unit.

To validate the regulatory role of 5-HMF in microglial M2 polarization, the targets of 5-HMF were predicted. The chemical structure of 5-HMF was downloaded from the online PubChem online database and is shown in Figure 2A. The potential targets of 5-HMF were predicted by the Swiss Target Prediction and PharmMapper databases, and 8 and 56 targets were obtained, respectively. By taking the intersection, 2 targets (MIF and PIM1) were obtained and displayed in a Venn diagram ( Figure 2B). As reported, MIF is associated with the pathogenesis of EAE [43]. Therefore, we investigated the interaction and function between MIF and 5-HMF. The 3D structure of MIF is shown in Figure 2C. Next, several possible molecular docking results were obtained from the SwissDock online server by uploading the gene information of MIF and 5-HMF. Using UCSF Chimera software, the optimal position information of the binding site was obtained; that is, in the best conformation, the oxygen atom O1 on the carbonyl group of 5-HMF and the N-terminal proline residue of the MIF monomer subunit (proline 1) formed a 2.202 Å hydrogen bond interaction. As shown in Figure 2D, the red, green, and blue colors represent the homotrimeric structure of MIF consisting of monomers with the same primary sequence, and the enlarged image represents the binding site of 5-HMF and MIF.

### MIF-CD74 complex is involved in ERK1/2-mediated microglial polarization

**Figure 3:**
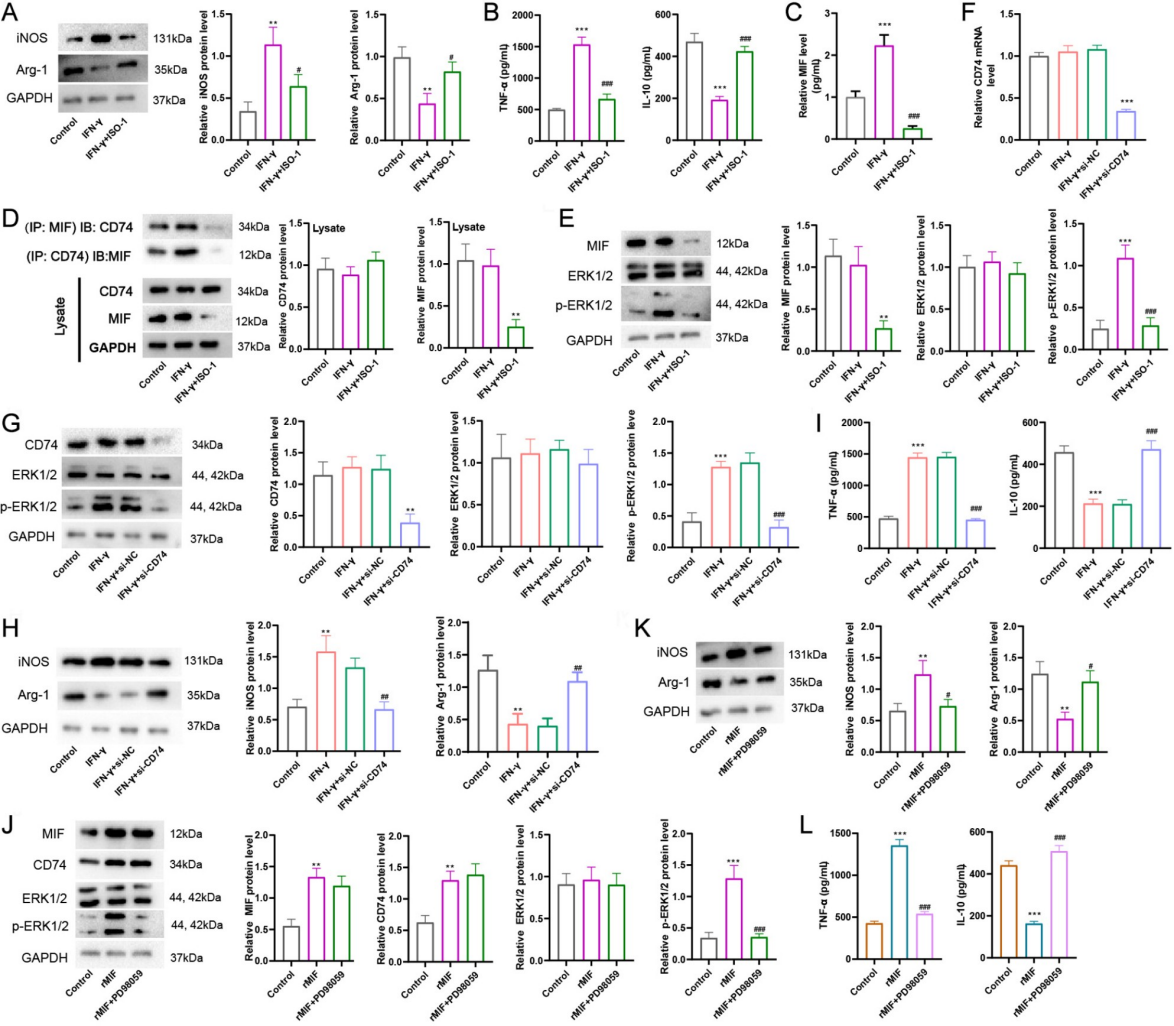
Figure 3 The MIF-CD74 complex is involved in ERK1/2-mediated microglial polarization BV2 cells were treated with IFN-γ (100 ng/mL) for 2 h, followed by treatment with the MIF inhibitor ISO-1 (50 μM) for 24 h. The cells were divided into 3 groups: control, IFN-γ, and IFN-γ+ISO-1. (A-C) The iNOS and Arg-1 protein levels in cells, TNFα and IL-10 concentrations in supernatants, and MIF in supernatants were measured. (D) A co-IP assay was performed to determine the interaction between MIF and CD74. (E) Western blot analysis of MIF, ERK1/2, and p-ERK1/2 protein levels. BV2 cells transfected with si-CD74 were treated with IFN-γ (100 ng/mL) for 24 h. Cells were divided into 4 groups: control, IFN-γ, IFN-γ+si-NC, and IFN-γ+si-CD74. CD74 mRNA level (F) and protein levels of CD74, ERK1/2, p-ERK1/2 (G), iNOS, Arg-1 (H), TNFα, and IL-10 (I) were measured. BV2 cells were supplemented with recombinant MIF (rMIF, 100 ng/mL) and cultured for 24 h, and then the ERK1/2 inhibitor PD98059 (10 μM) was added and incubated for 24 h. Cells were divided into control, rMIF, and rMIF+PD98059 groups. Protein levels of MIF, CD74, ERK1/2, p-ERK1/2 (J), iNOS, Arg-1 (K), TNFα, and IL-10 (L) were measured. GAPDH was used as an internal control. Statistical analysis was performed by one-way ANOVA followed by Tukey’s post hoc test. Data are from three independent experiments. ** P<0.01, *** P<0.001 vs control; #P<0.05, ##P<0.01, ###P<0.001 vs IFN-γ or IFN-γ+si-NC or rMIF.

Previous reports have described that MIF can interact with CD74 to alter the phosphorylation of ERK1/2 and regulate the activation of Schwann cells [28] and chondrocytes [29]. We investigated whether the MIF-CD74 interaction regulates microglial M1 polarization via activation of the ERK1/2 pathway. BV2 cells were stimulated with IFN-γ with or without an MIF inhibitor (ISO-1). As shown in Figure 3A,B, ISO-1 reduced the iNOS protein level in cells and the TNF-α level in supernatants, whereas it restored the Arg-1 and IL-10 levels. In addition, IFN-γ induced the upregulation of MIF in supernatants, whereas ISO-1 reduced this level ( Figure 3C). The results from the co-IP assay showed a greater interaction of MIF and CD74 in the IFN-γ treatment group than in the IFN-γ+ISO-1 group ( Figure 3D). IFN-γ treatment also induced the upregulation of p-ERK1/2, and ISO-1 reduced this level ( Figure 3E). These data suggested that blocking MIF activity contributed to the decrease in M1 polarization and the inflammatory response.

To further understand whether CD74 is involved in M1 polarization, BV2 cells were transfected with si-CD74 followed by IFN-γ stimulation. The transfection efficiency was determined by qRT-PCR and western blot analysis ( Figure 3F,G). si-CD74 transfection reduced the p-ERK1/2 level, which was elevated by IFN-γ ( Figure 3G). si-CD74 transfection also reduced iNOS and TNF-α levels and increased Arg-1 and IL-10 levels, indicating that *CD74* silencing inhibited M1 polarization, promoted M2 polarization, and enhanced the anti-inflammatory response ( Figure 3H,I). Meanwhile, rMIF significantly increased the levels of MIF, CD74, p-ERK1/2, iNOS, and TNF-α, whereas PD98059 markedly decreased the levels of p-ERK1/2, Arg-1, and IL-10 ( Figure 3J,K). Taken together, these data indicated that MIF-CD74 regulated microglial polarization and inflammation through the ERK1/2 pathway.

### 5-HMF inhibits M1 polarization by blocking the binding of MIF with CD74

**Figure 4:**
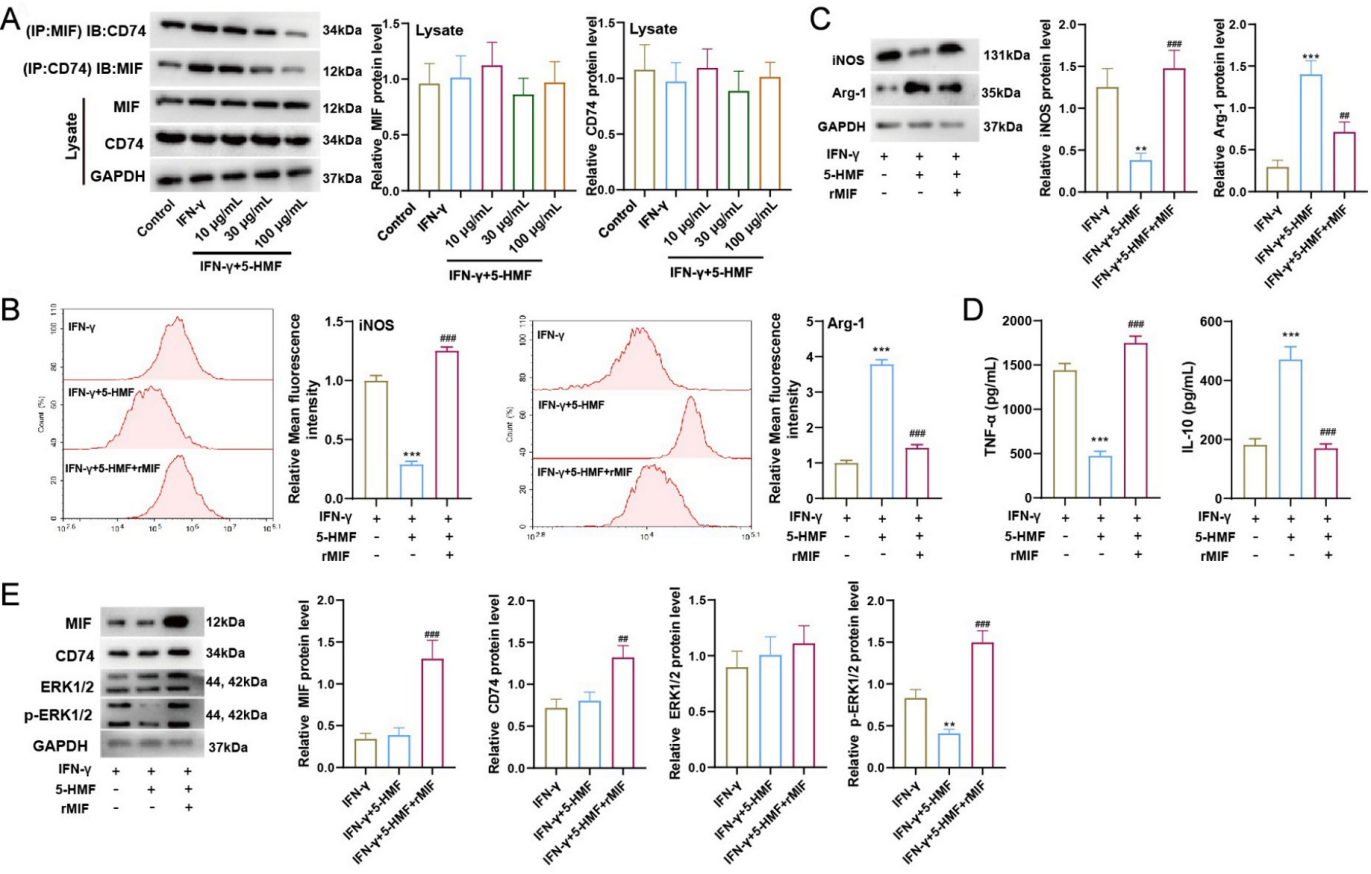
Figure 4 5-HMF inhibits M1 polarization by blocking the binding of MIF with CD74 IFN-γ (100 ng/mL) was added to BV2 cells and cultured for 2 h, and 5-HMF at 10, 30, and 100 μg/mL was used to stimulate cells for 24 h. (A) Using anti-MIF or anti-CD74, a co-IP assay was performed to confirm the interaction between MIF and CD74. The protein level of CD74 or MIF in the immunoprecipitations was detected by western blot analysis. Then, after IFN-γ (100 ng/mL) stimulation for 2 h, BV2 cells were treated with 5-HMF (100 μg/mL) with or without rMIF (100 ng/mL) treatment for 24 h. The cells were divided into 3 groups: IFN-γ, IFN-γ+5-HMF, and IFN-γ+5-HMF+rMIF. (B) Flow cytometry analysis. Relative iNOS-positive cells and Arg-1-positive cells were evaluated by determination of the mean fluorescence intensity. iNOS and Arg-1 levels (C), TNF-α and IL-10 levels in cell supernatants (D) and MIF, CD74, ERK1/2, and p-ERK1/2 levels in BV2 cells (E) were measured. GAPDH was used as an internal control. Statistical analysis was performed by one-way ANOVA followed by Tukey’s post hoc test. Data are from three independent experiments. ** P<0.01, *** P<0.001 vs IFN-γ; ##P<0.01, ###P<0.001 vs IFN-γ+5-HMF.

Several studies have reported that the proline residue PRO is located between the monomeric subunits of MIF [44], and the proline residue PRO can bind to the CD74 receptor [45]. We speculated that 5-HMF may block the binding of MIF with CD74. BV2 cells were stimulated with IFN-γ followed by 5-HMF treatment. According to the Co-IP assay results, the interaction of MIF with CD74 was increased when treated with IFN-γ, while this interaction gradually decreased with increasing doses of 5-HMF ( Figure 4A). Next, BV2 cells were stimulated with IFN-γ followed by 5-HMF treatment with or without rMIF. Flow cytometry results showed that 5-HMF reduced iNOS-positive cells and enhanced Arg-1-positive cells, while these results were reversed by rMIF ( Figure 4B). In addition, 5-HMF treatment reduced iNOS protein levels in IFN-γ-stimulated cells and TNF-α levels in supernatants, whereas these effects were abolished by rMIF ( Figure 4C,D). Furthermore, IFN-γ induced the downregulation of p-ERK1/2, whereas rMIF increased the protein levels of CD74 and p-ERK1/2 ( Figure 4E).

### 5-HMF ameliorates EAE symptoms by blocking the MIF-CD74 interaction

**Figure 5:**
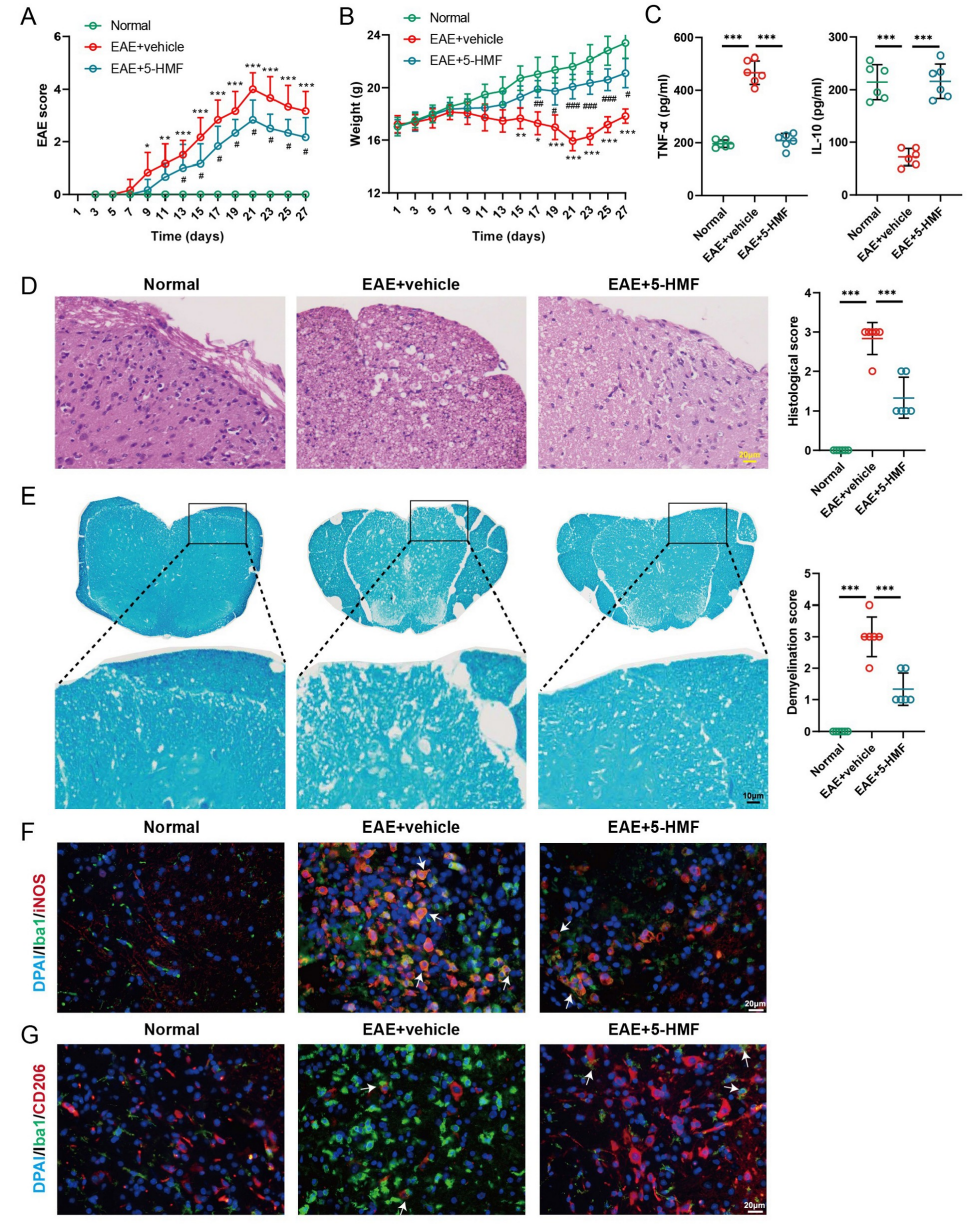
Figure 5 5-HMF ameliorates EAE symptoms by blocking the MIF-CD74 interaction An EAE mouse model was established, and the model mice were treated with 5-HMF (12 mg/kg) by intraperitoneal injection daily from day 3 to day 27 after modelling. Animals were divided into 3 groups ( n=6 per group): normal, EAE+vehicle, and EAE+5-HMF. (A,B) The EAE scores and weights of the mice were measured and statistically analysed. (C) TNFα and IL-10 levels in mouse serum were detected. (D,E) Hematoxylin-eosin (H&E) and Luxol fast blue (LFB) staining of the spinal cords of mice. Histologic scores and demyelination scores were analysed. (F,G) Immunofluorescence staining of the lumbar spinal cord. Scale bar: 20 μM.Double staining for Iba1 and iNOS or CD206 was performed. Statistical analysis was performed by one-way ANOVA followed by Tukey’s post hoc test. * P<0.05, ** P<0.01, *** P<0.001 vs normal or EAE+vehicle; #P<0.05, ##P<0.01, ###P<0.001 vs EAE+vehicle.

**Figure 6:**
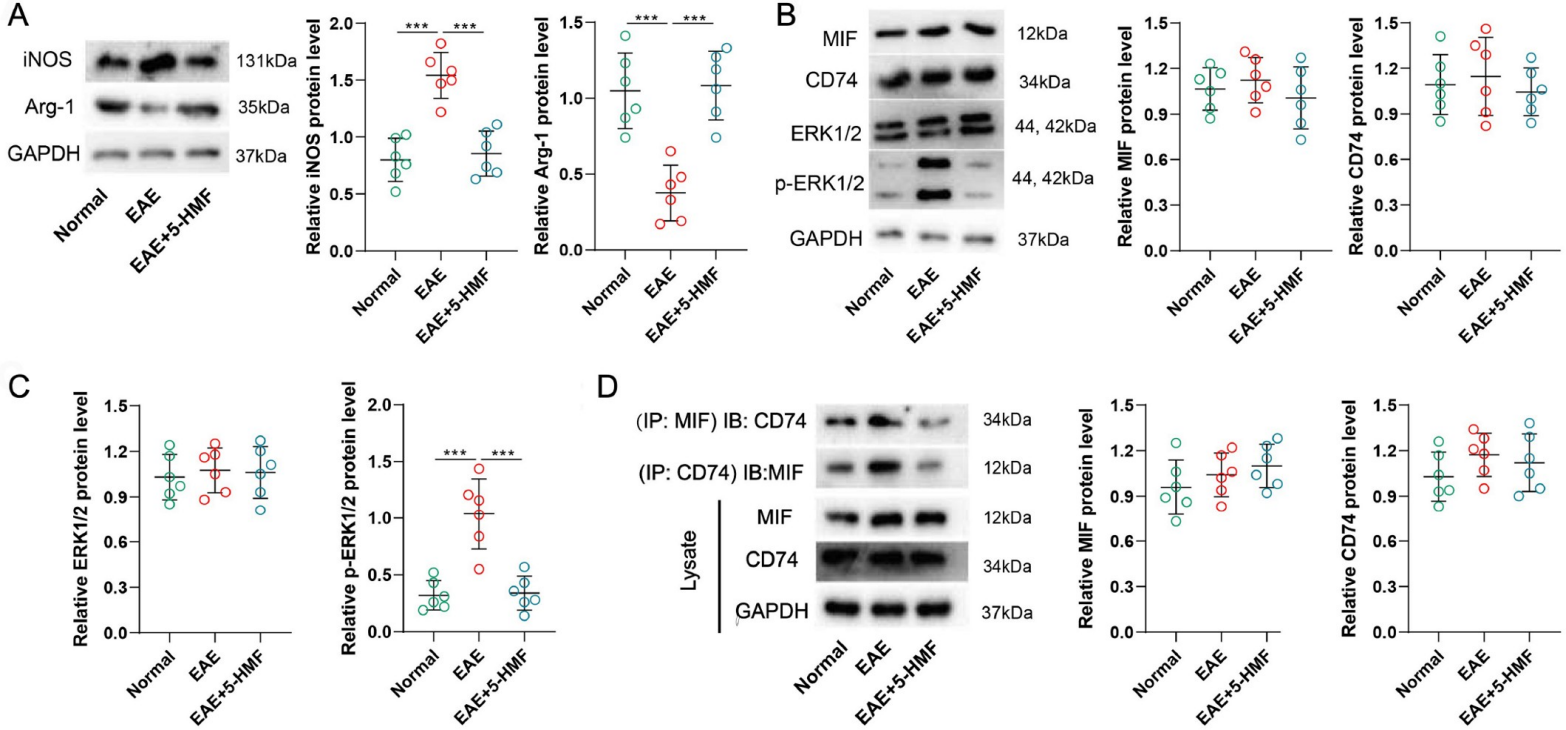
Figure 6 5-HMF ameliorates EAE symptoms by blocking the MIF-CD74 interaction An EAE mouse model was established, and the model mice were treated with 5-HMF (12 mg/kg) by intraperitoneal injection daily from day 3 to day 27 after modelling. Animals were divided into 3 groups ( n=6 per group): normal, EAE+vehicle, and EAE+5-HMF. (A-C) Protein levels of iNOS, Arg-1, MIF, CD74, ERK1/2, and p-ERK1/2. (D) The co-IP assay was performed using anti-CD74 or anti-MIF. The protein level of CD74 or MIF in the immunoprecipitations was assessed by western blot analysis. GAPDH was used as an internal control. Statistical analysis was performed by one-way ANOVA followed by Tukey’s post hoc test. *** P<0.001.

To determine the effect of 5-HMF *in vivo*, C57BL/6 mice were induced to establish an EAE model followed by intraperitoneal injection of 5-HMF. Compared with the normal group, the model mice exhibited EAE symptoms from day 7, showing tail paralysis, bradykinesia, and mild abnormal gait. The EAE scores increased and peaked on day 21. Importantly, 5-HMF treatment significantly improved the severity of EAE symptoms ( Figure 5A). From day 7, the weight of mice in the EAE group was much lower than that of the control group, and the EAE mice reached the lowest weight on day 21. Similarly, the 5-HMF treatment significantly improved this growth retardation ( Figure 5B). In mouse serum, EAE induced the upregulation of TNF-α and downregulation of IL-10, whereas 5-HMF reversed these changes ( Figure 5C). We next examined the effects of 5-HMF on inflammation and demyelination by histologic analysis. H&E and LFB staining of the lumbar spinal cords showed that EAE induced white matter injury, infiltrated inflammatory cells, and demyelination, whereas 5-HMF treatment stopped the worsening of these inflammatory lesions ( Figure 5D,E). Furthermore, according to the immunofluorescence staining results, EAE promoted the activation of M1 polarization, showing an increased level of iNOS in Iba1 ^+^ cells, while 5-HMF treatment reversed these results. Meanwhile, EAE reduced the level of CD206 in Iba1 ^+^ cells, and these effects were abolished by 5-HMF ( Figure 5F,G). In addition, we found that 5-HMF reversed the protein levels of iNOS, Arg-1, and p-ERK1/2 in the EAE group ( Figure 6A–C). Finally, the interaction of MIF with CD74 was examined, and the co-IP results showed that the MIF-CD74 interaction was increased in the EAE group, whereas it was reduced by 5-HMF ( Figure 6D). Collectively, these data suggested that 5-HMF ameliorated the severity of EAE, inflammation, and demyelination by blocking the MIF-CD74 interaction.

## Discussion

**Figure 7:**
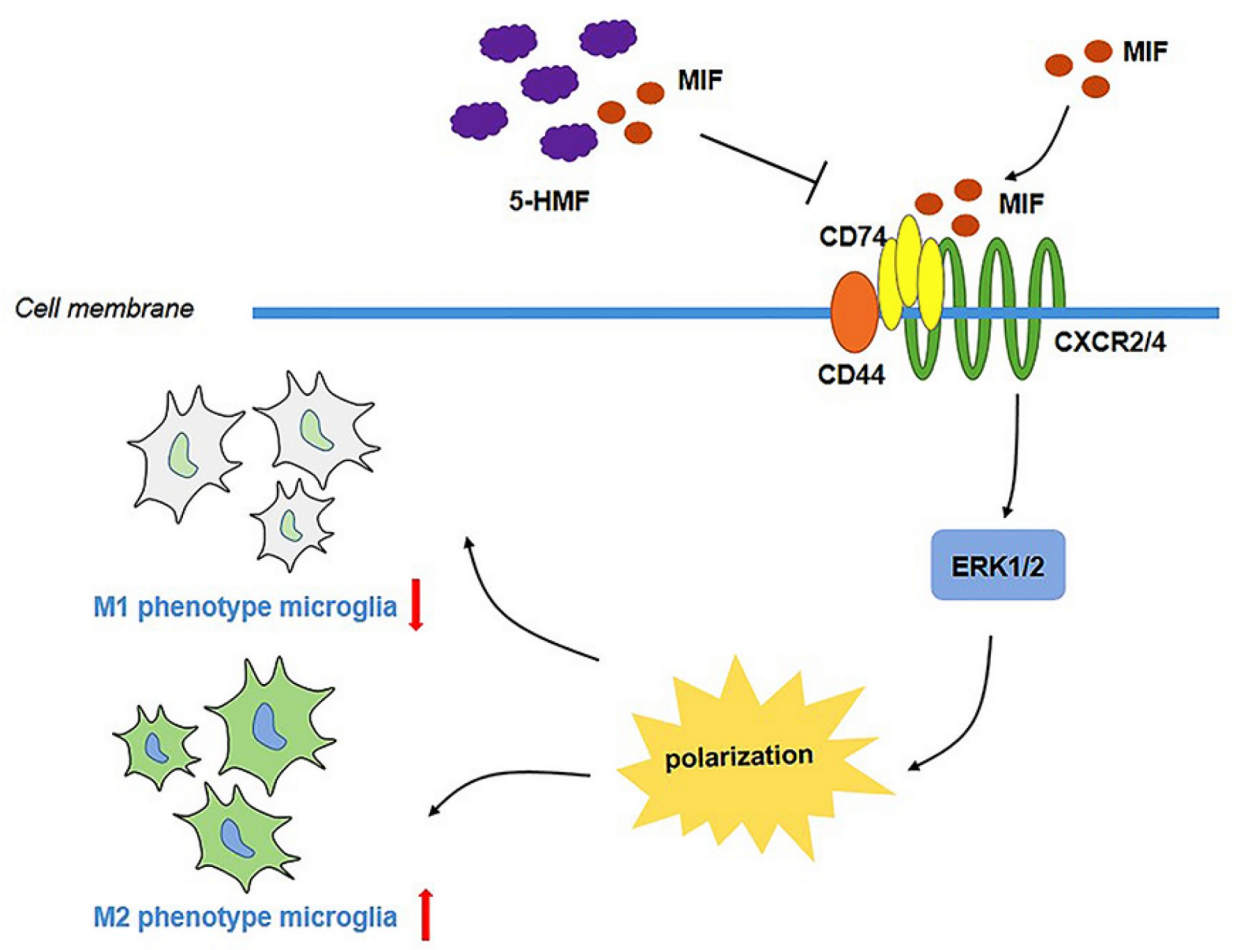
Figure 7 Schematic of the signaling pathways by which 5-HMF affects microglial polarization in EAE BV2 cells were stimulated with IFN-γ to induce the neuroinflammatory model. The effect of 5-HMF or MIF on microglial M1/2 polarization and the interactions of 5-HMF and MIF or MIF and CD74 were confirmed. Finally, we validated whether the MIF-CD74 complex involves 5-HMF in affecting cell polarization and inflammation.

Growing evidence has demonstrated the significant effect of the herbal medicine Bu-Shen-Yi-Sui capsule in ameliorating MS [ 10, 46] . In this study, we further investigated the effect of the component 5-HMF on inflammation and demyelination in the CNS of MS. The results showed that 5-HMF facilitated microglial M2 polarization and attenuated the inflammatory response. Network pharmacology-based prediction and molecular docking analysis were used to validate the binding between 5-HMF and MIF. Further data showed that the MIF-CD74 complex was involved in the regulation of microglial polarization by altering the phosphorylation of ERK1/2, and 5-HMF prevented M1 polarization by inhibiting the MIF-CD74 interaction ( Figure 7). *In vivo*, 5-HMF ameliorated the severity of EAE and reduced inflammation and demyelination.

In MS, microglia are activated and release inflammatory cytokines, resulting in nerve damage; therefore, inhibition of microglial overactivation may be a novel approach for MS treatment [47]. Microglial M1/M2 polarization had a significant effect on neuroinflammation during EAE. Microglial M1 polarization produces high levels of oxidant metabolites, leading to CNS dysfunction [48], whereas the M2 phenotype induces tissue repair in the CNS by secreting cytokines and expressing anti-inflammatory-related receptors [49]. In our study, 5-HMF markedly increased microglial M2 polarization and anti-inflammatory cytokine production and reduced microglial M1 polarization and proinflammatory cytokine levels, as shown by the increased Arg-1 and IL-10 levels and decreased iNOS and TNF-α levels in BV2 cells. EAE is the most commonly used potent and preclinical murine model of human MS and shares many pathologic and histologic similarities with MS [ 5, 50] . The *in vivo* data showed that 5-HMF treatment significantly reduced EAE scores and increased the body weight of the mice. Through H&E and LFB staining observations, EAE was found to induce white matter injury, infiltrated inflammatory cells, and demyelination in the spinal cord, whereas 5-HMF treatment attenuated these inflammatory lesions. In addition, we confirmed M1- and M2-polarized microglia by immunofluorescence using iNOS and Arg1 as markers. Iba1 is a microglia/macrophage-specific calcium-binding protein that is commonly used to detect activated microglia [51]. 5-HMF treatment resulted in significantly increased expression of Arg1 in Iba1 ^+^ cells, while iNOS levels were markedly reduced in Iba1 ^+^ cells from EAE mice. Similarly, 5-HMF treatment induced the upregulation of IL-10 but downregulated TNF-α. These data demonstrated the neuroprotective effects of 5-HMF in EAE. We also isolated and cultured primary microglia and confirmed that 5-HMF promoted the polarization of primary microglia toward the M2 phenotype and attenuated the inflammatory response ( Supplementary Figure S1). However, because the BV2 cell line is more stable, we performed subsequent experiments to investigate the mechanism using BV2 cells.

According to the results of the Swiss Target Prediction and PharmMapper database, MIF was identified to interact with 5-HMF, showing that the oxygen atom O1 on the carbonyl group of 5-HMF and the N-terminal proline residue of the MIF monomer subunit (proline 1) formed a 2.202 Å hydrogen bond interaction. MIF is a pleiotropic cytokine that activates intracellular signaling of target cells, and it is also considered to be a key regulator of the inflammatory response via interaction with the CD74 receptor to activate the ERK signaling pathway [28]. CD74 is a type II transmembrane protein, and MIF/CD74 signaling affects M1 macrophage polarization in obesity-related adipose tissue inflammatory disease [27]. Our results showed that blocking MIF activity prevented microglial M1 polarization, the proinflammatory response, and ERK1/2 phosphorylation. The co-IP assay confirmed the interaction between MIF and CD74. Meanwhile, *CD74* silencing reduced ERK1/2 phosphorylation, inhibited microglial M1 polarization, promoted microglial M2 polarization, and enhanced the anti-inflammatory response. Simultaneous treatment with recombinant MIF and ERK1/2 inhibitor rMIF significantly increased the protein levels of CD74, p-ERK1/2, iNOS, and TNF-α, whereas ERK1/2 inhibitor markedly decreased the levels of p-ERK1/2, Arg-1, and IL-10. Based on these findings, we suggest that MIF/CD74 regulates microglial polarization and inflammation through the ERK1/2 pathway.

To determine the regulatory role of 5-HMF in MIF/CD74 signaling and the downstream effects, the function of 5-HMF on the MIF and CD74 interaction was examined. As shown by the co-IP assay, the MIF-CD74 interaction gradually decreased with increasing concentrations of 5-HMF. Furthermore, rMIF treatment reversed the effect of 5-HMF on microglial M1/2 polarization, inflammatory cytokine production, and ERK1/2 phosphorylation. More importantly, the *in vivo* data also confirmed the interaction of MIF with CD74, showing that EAE induced an increase in the MIF-CD74 interaction, while 5-HMF inhibited this change. Consistent with the *in vitro* data, 5-HMF treatment reversed the protein levels of MIF, CD74, and p-ERK1/2 in EAE mice. These data further suggest the potential mechanism by which 5-HMF promotes M2 polarization in EAE mice, which is related to the alteration of MIF/CD74 signaling. Similar to our results, some studies emphasized the protective role in hypoxic neurogenic diseases [ 52, 53] . In addition, Benedek *et al*. [26] demonstrated that recombinant TCR ligands (RTLs) inhibited MIF/CD74 binding and inflammation in EAE, which showed a consistent research strategy. These studies provided the scientific basis and theoretical support for our work. Ghoochani *et al*. [22] demonstrated that MIF-CD74 inhibited glioma-associated microglial M1 polarization via ERK1/2 signaling and promoted glioma growth. In the present study, based on network pharmacology analysis and molecular docking predictions, we confirmed that 5-HMF inhibited microglial M1 polarization by binding to MIF and thus inhibiting the MIF-CD74 interaction, which is the novelty of this study. Although the MIF/CD74/ERK1/2 pathway regulating microglial polarization is not new, we believe that the validation of this pathway regulating microglial polarization is necessary to clarify the mechanism of 5-HMF-mediated microglial M1 polarization.

A block in the M1 to M2 switch may contribute to remyelination failure in chronic inactive MS lesions [49]. Microglia with the M2 phenotype can ameliorate inflammation-induced demyelination and promote EAE recovery [54]. In this study, we demonstrated that 5-HMF inhibited ERK1/2 phosphorylation levels through competitive binding of MIF in microglia, resulting in increased M2 polarization accompanied by decreased TNF-α secretion; *in vivo* experiments confirmed that 5-HMF administration improved EAE symptoms in mice, including decreased EAE score, reduced spinal cord proinflammatory factor levels, and decreased demyelination area. In addition, our results showed increased levels of phosphorylated ERK1/2 in the spinal cord of the EAE model, which is consistent with the study of Zhang *et al*. [55]. Our data suggest that 5-HMF ameliorates EAE symptoms in mice, and its mechanism may be through the MIF/CD74/ERK1/2 signaling axis to drive microglia to an M2 phenotype. However, for *in vivo* experiments, we cannot exclude the effect of 5-HMF on other cells, such as astrocytes and neuronal cells. This part still needs further investigation.

Collectively, our data suggest the critical involvement of 5-HMF in microglial M1/2 polarization, inflammation, and demyelination via regulation of the MIF/CD74 axis in the CNS of EAE. Understanding the molecular mechanism underlying inflammation and demyelination may help provide novel therapeutic targets to prevent the development of MS.

## Supporting information

494FigS1

## Supplementary Data

Supplementary data is available at *Acta Biochimica et Biphysica Sinica* online.
