# Supplementary material for: 5-HMF attenuates inflammation and demyelination in experimental autoimmune encephalomyelitis mice by inhibiting the MIF-CD74 interaction: Role of 5-HMF in EAE mice
Source: Acta Biochim Biophys Sin (Shanghai). 2023 Jul 10;55(8):1222–33. doi: 10.3724/abbs.2023105 (PMC10448060; doi:10.3724/abbs.2023105)
Supplement: 494FigS1 [file 494FigS1.pdf]

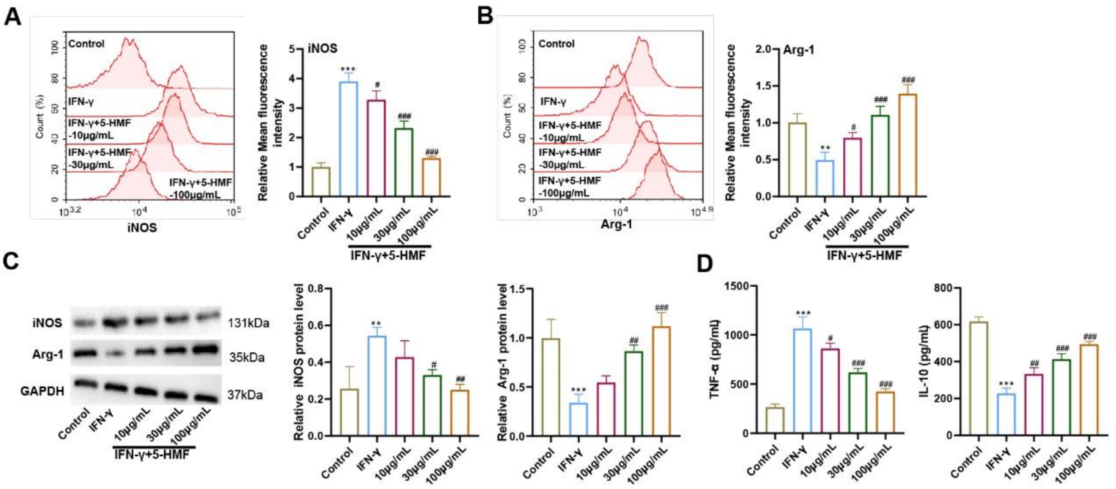

Supplementary Figure S1. 5-HMF facilitates IFN-γ-stimulated microglial M2 polarization and attenuates the inflammatory response Primary microglia were stimulated with IFN-γ (100 ng/mL) for 2 h followed by 5-HMF treatment at concentrations of 10, 30, and 100 μg/mL. Cells were grouped as follows: control, IFN-γ, and IFN-γ + 5-HMF (10, 30, and 100 μg/mL). (A,B) Flow cytometry analysis. The iNOS-positive cells and Arg-1-positive cells were analysed. The mean fluorescence intensity of each condition was analysed. (C, D) Protein levels of iNOS and Arg-1 in cells and concentrations of TNF-α and IL-10 in cell supernatants were measured by Western blot and ELISA. GAPDH was used as an internal control. Statistical analysis was performed by one-way ANOVA followed by Tukey's post hoc test. Each experiment was performed in triplicate independently. $^{**}P < 0.01$, $^{***}P < 0.001$ vs control; $^{\#}P<0.05$, $^{\#\#}P<0.01$, $^{\#\#\#}P < 0.001$ vs IFN-γ.
